# Mapping of conserved immunodominant epitope peptides in the outer membrane porin (Omp) L of prominent *Enterobacteriaceae* pathogens associated with gastrointestinal infections

**DOI:** 10.1186/s43141-023-00622-6

**Published:** 2023-11-28

**Authors:** Harish Babu Kolla, Shivakiran Satyanarayan Makam, Prakash Narayana Reddy

**Affiliations:** 1 Department of Biotechnology, Vignan’s Foundation for Science, Technology and Research, Vadlamudi, Guntur, 522213 Andhra Pradesh India; 2 SKU Confederation, Atal Incubation Center, Sri Krishnadevaraya University, Anantapur, 515003 Andhra Pradesh India; 3 Department of Microbiology, Dr. V.S. Krishna Government Degree & PG College, Maddilapalem, Visakhapatnam, 530013 Andhra Pradesh India

**Keywords:** Outer membrane porin (Omp) L, Sequence homology, B-cell epitopes, Epitope conservancy

## Abstract

**Background:**

Members of *Enterobacteriaceae* such as *Escherichia coli* O 157:H7, *Salmonella* sp., *Shigella* sp., *Klebsiella* sp., and *Citrobacter freundii* are responsible for the outbreak of serious foodborne illness and other mucosal infections across the globe. The outer membrane proteins (OMPs) of *Enterobacteriaceae* are highly immunogenic in eliciting immune responses against pathogens. Moreover, the OMPs are highly conserved in the *Enterobacteriaceae* family. Sequence homology in the OMPs will ensure the presence of conserved immunodominant regions with predominant epitopes. The OmpL is such an immunogen that is highly conserved among the *Enterobacteriaceae* pathogens. In this study, we performed computational analysis on the outer membrane porin (Omp) L of prominent *Enterobacteriaceae* pathogens.

**Results:**

Multiple sequence and structural alignment analysis have revealed that the OmpL protein is highly conserved among the selected *Enterobacteriaceae* pathogens. This amount of sequence and structural homology uncovered the conserved antibody binding B-cell epitopes in the OmpL protein. The B-cell epitopes predicted in the OmpL of *Salmonella typhimurium* are highly conserved among the other *Enterobacteriaceae* pathogens.

**Conclusion:**

In conclusion, these conserved B-cell epitopes will vouch for the generation of heterologous humoral immune response in conferring cross protection against the *Enterobacteriaceae* pathogens and control their outbreaks across the globe.

## Background

Members of the *Enterobacteriaceae* family cause serious foodborne illnesses across the globe. It has remained a serious threat to the public in both developed and developing countries. The *Enterobacteriaceae* members contaminate food and water and are responsible for the primary cause of foodborne outbreaks around the world. Some of the *Enterobacteriaceae* members such as *Escherichia coli*, *Salmonella* sp., and *Shigella* sp. are the major foodborne pathogens [1]. These enteric pathogens are mainly associated with serious food poisoning outbreaks. Moreover, these bacteria impart serious concerns to public health in Asian and African countries [1]. Being an opportunistic pathogen, many serotypes of *E. coli* have emerged over time as an evolutionary event. According to the World Health Organization (WHO), the Shige toxin (Stx)-producing *E. coli* is associated with serious foodborne outbreaks (https://www.who.int/news-room/fact-sheets/detail/e-coli). The O157:H7 serotype of *E. coli* is a representative pathogroup of the Stx-producing *E. coli* and causes hemolytic uremic syndrome (HUS) and hemorrhagic colitis (HC) [2]. Besides, *Shigella* sp. and *Salmonella* are other *Enterobacteriaceae* members with high virulence. *Shigella* causes shigellosis, an acute intestinal infection that is caused by the consumption of contaminated food and water. The species of *Shigella*, *boydii*, *sonnei*, *flexneri*, and *dysenteriae* are known to cause bacillary dysentery even in very low CFU counts [3]. On the other hand, serovars of *Salmonella enterica* subsp. *enterica* like *typhimurium*, *enteritidis*, *typhi*, and *paratyphi* (A, B, and C) cause various distinct types of infections, these being salmonellosis, typhoid (by typhi), and paratyphoid fever (by paratyphi) [4]. Apart from these pathogens, *Citrobacter freundii* and *Klebsiella* sp. (mainly *pneumoniae* and *oxytoca*) are also associated with gastrointestinal and other mucosal infections [5–7].

In this context, vaccination and other prophylactic therapies are the major solutions to control disease outbreaks mediated by these infectious pathogens. Several studies have proven the immunoprophylactic potential of outer membrane proteins (OMPs) of gram-negative bacteria [8–11]. Above and beyond, OMPs are highly immunogenic by the virtue of the exposed immunodominant epitopes and therefore would be effective targets for vaccine development and passive immune therapy. According to Meenakshi et al., the OMPs of *Salmonella* have been known to play a crucial role in eliciting immune response [12]. Additionally, the OMPs are highly conserved across the proteobacteria members. For instance, a recent study by Liu et al. demonstrated that the OmpC of *Salmonella typhi* and OmpK36 of *Klebsiella pneumoniae* share greater sequence identity, and cross-reactive neutralizing antibodies were identified in mice sera [11].

In the early years of the decade, outer membrane porin (Omp) L which has been investigated by Yang et al. showed 100% protection in mice against salmonellosis and the other *Enterobacteriaceae* members such as *Klebsiella oxytoca*, *E. coli* O157:H7, *Shigella*, and *Citrobacter koseri* formed as a single cluster in the phylogenetic tree of OmpL [10]. Because the protection is high and the sequence homology is seen in OmpL among the *Enterobacteriaceae*, there is a fair chance of the presence of conserved epitopes which can be further characterized and used in developing a broad-spectrum vaccine against enteric pathogens. This has motivated us to unravel the sequence homology which exemplifies the conserved B-cell epitopes in OmpL protein among the pathogenic *Enterobacteriaceae* members which can lead to antibody-mediated cross-reactivity and neutralization. We employed various in silico tools and techniques of immunoinformatics for the identification of conserved immunodominant B-cell epitopes in the OmpL of the selected *Enterobacteriaceae* members [13–15] vaccine candidate against *Enterobacteriaceae *members.

## Methods

### Sequence retrieval

The complete amino acid sequence of the OmpL protein (230 amino acids) of *Salmonella typhimurium* bearing an accession ID: Q9L7R3 was retrieved from the UniProt resource (https://rest.uniprot.org/uniprotkb/Q9L7R3.fasta). The signal peptide ranging from 1 to 20 amino acids in the OmpL protein was excluded from the analysis. The core protein region of 21–230 amino acids was subjected to the ProtParam tool (https://web.expasy.org/protparam/) for its physicochemical parameter analysis and then was used for BLASTp analysis to mine the sequences of other *Enterobacteriaceae *pathogens *E. coli* O 157:H7, *Shigella* sp., *Salmonella* sp., *Klebsiella* sp., and *C. freundii* (Table 1). These mined sequences were downloaded from NCBI and saved in FASTA format with an extension “.fasta” or “.fa.”

**Table 1:** Accession IDs of the mined amino acid sequences of OmpL protein

Strain name	Accession ID
*Salmonella typhimurium* ATCC 700720	Q9L7R3
*Salmonella enterica* subsp. enterica serovar Enteritidis	EEA2672579.1
*Salmonella enterica* subsp. enterica serovar Typhi	EHK2171593.1
*Salmonella enterica* subsp. enterica serovar Paratyphi A	EGI6603164.1
*Salmonella enterica* subsp. enterica serovar Paratyphi B	EDC2010720.1
*Salmonella enterica* subsp. enterica serovar Paratyphi C	HCB5057200.1
*Escherichia coli* O157:H7	EFE2127272.1
*Citrobacter freundii*	MCI1670881.1
*Klebsiella pneumoniae*	MCM5985717.1
*Klebsiella oxytoca*	RFP39752.1
*Shigella boydii*	EFZ2304433.1
*Shigella flexneri*	EFZ8854897.1
*Shigella sonnei*	EFZ4853893.1
*Shigella dysenteriae*	EJE7370028.1

### Protein modeling and refinement

Protein structures of OmpL of *E. coli* O 157:H7, *Shigella* sp., *Salmonella* sp., *Klebsiella* sp., and *C. freundii* were modeled and refined to the native biological conditions for predicting the discontinuous B-cell epitopes from the modeled 3-D structures of OmpL protein. The three-dimensional structures of core protein region (21–210 amino acids) of OmpL proteins were predicted from the amino acid sequences with Roberta server (https://robetta.bakerlab.org/) [16]. Further, the predicted structures were refined to improve their structural quality and bring the structures close to the experimental precision for the efficient prediction of discontinuous B-cell epitopes.

### Multiple sequence and structural alignment

All the amino acid sequences retrieved through protein mining with BLASTp were used for our analysis. Multiple sequence alignment (MSA) was performed for the retrieved amino acid sequences to determine the sequence homology in the OmpL protein among *Enterobacteriaceae *members. An online program Clustal Omega (https://www.ebi.ac.uk/Tools/msa/clustalo/) was used for aligning the amino acid sequences of OmpL. The output file was saved in MEGA format with an extension “.meg” or “.mega.” The amino acid conservancy was determined in the alignment file of OmpL protein with the help of MEGA-X software. The structural alignment for the predicted structures was carried out using PDB pairwise structure alignment resource (https://www.rcsb.org/alignment).

### B-cell epitopes

Firstly, the linear and discontinuous B-cell epitopes present in core region (21aa–230aa) of OmpL protein of *S. typhimurium* were predicted. Later, these predicted B-cell epitopes were analyzed for their conservancy among the selected *Enterobacteriaceae* pathogens. For the prediction of linear B-cell epitopes, we used an online tool BepiPred (https://services.healthtech.dtu.dk/service.php?BepiPred-2.0) at a default threshold score of 0.5 where the peptide regions in OmpL protein of *S. typhimurium* with prediction scores greater than or equal to 0.5 were determined as epitopes [17]. The discontinuous B-cell epitopes were predicted from the 3-D structures of the OmpL proteins of *E. coli* O 157:H7, *Shigella* sp., *Salmonella* sp., *Klebsiella* sp., and *Citrobacter freundii* with DiscoTope tool in IEDB server (http://tools.iedb.org/discotope/) [18].

### Epitope conservancy

The B-cell and T-cell epitopes in OmpL protein of *S. typhimurium* were analyzed for their conservancy among the selected *Enterobacteriaceae* pathogens. For this, the predicted B-cell and T-cell epitopes in OmpL protein of *S. typhimurium* were searched in the alignment file of OmpL, and epitope conservancy among all the *Enterobacteriaceae* pathogens was represented in percentage.

## Results

### Amino acid conservancy in OmpL

**Fig. 1:**
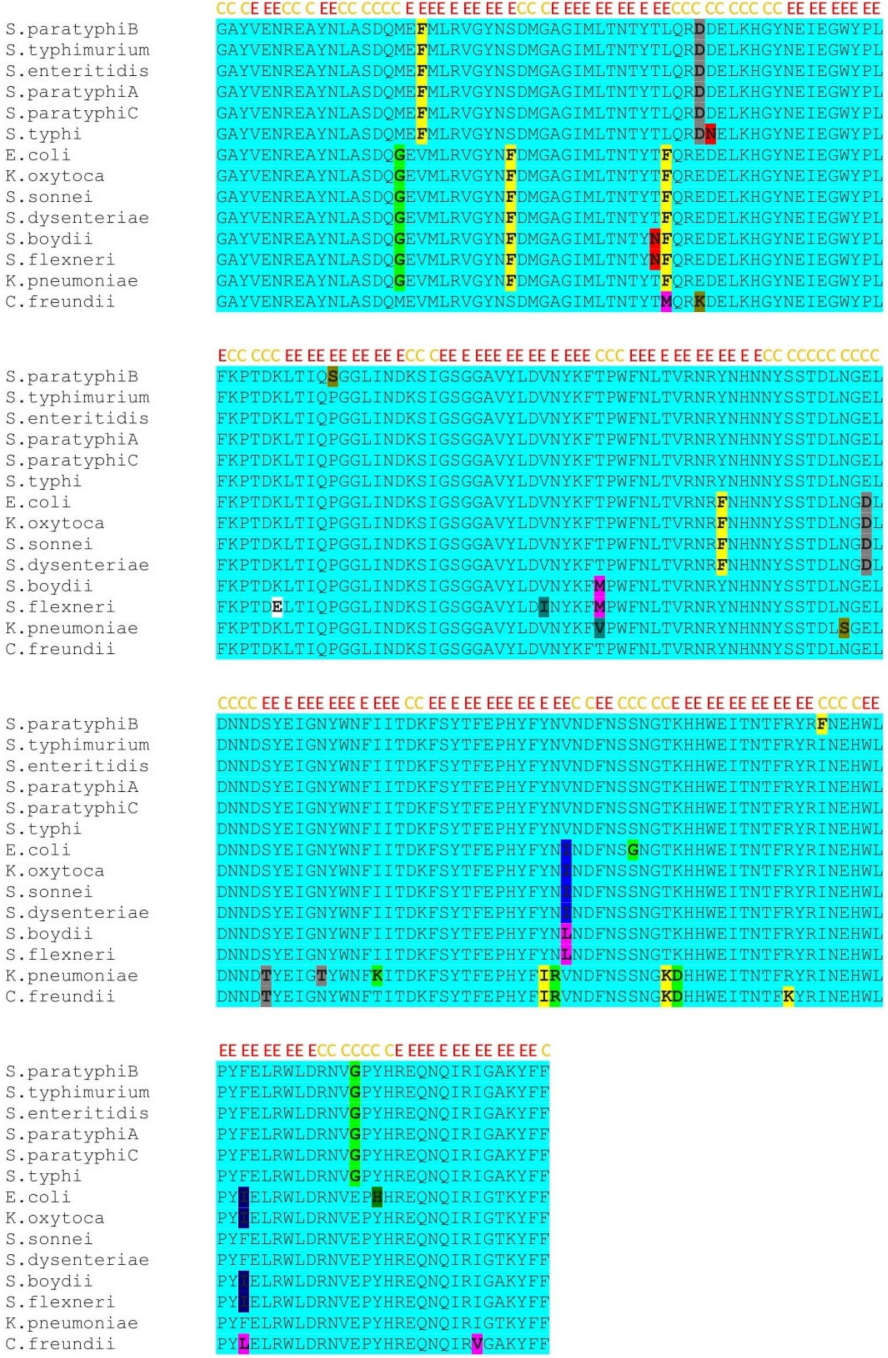
Amino acid sequence conservation in the OmpL protein of *Enterobacteriaceae* pathogens. The OmpL protein is highly conserved in the selected *Enterobacteriaceae* pathogens with a sequence identity of ~ 82% (conserved regions are highlighted in the yellow regions). The coils in protein structure are represented with “C” and strands are highlighted as “E” on the sequence in alignment

The OmpL is a 25-kDa membrane protein with an isoelectric point of 5.46. Also, it is a stable protein with an instability index of 20.98 (proteins with an instability index of less than 40 are found to be stable). The MSA analysis revealed that the OmpL protein is highly conserved among the selected *Enterobacteriaceae* pathogens *E. coli* O157:H7, *Shigella* sp., *Salmonella* sp., *Klebsiella* sp., and *Citrobacter freundii* (Fig. 1). Table 2 shows the % identity matrix of OmpL among the *Enterobacteriaceae* members. A total of 172 amino acid residues are conserved out of the 210 amino acids with variations in only 38 amino acids in the core protein of OmpL of the selected *Enterobacteriaceae* pathogens. This amino acid conservancy has shown that the OmpL protein has a higher sequence identity of ~ 82% among *Enterobacteriaceae* members which signifies the presence of conserved immunodominant epitopes to confer cross-protection against these pathogens certainly.

**Table 2:** Percent identity matrix representing the sequence homology of the OmpL protein of *Enterobacteriaceae* pathogens

	***K. oxytoca***	***C. freundii***	***K. pneumoniae***	***S. typhimurium***	***S. enteritidis***	***S. typhi***	***S. paratyphi* A**	***S. paratyphi* B**	***S. paratyphi* C**	***S. flexneri***	***S. boydii***	***S. sonnei***	***S. dysenteriae***	***E. coli* O157:H7**
*K. oxytoca*	100	90.95	90.95	90.00	90.00	90.00	90.00	90.00	90.00	86.67	87.62	88.10	88.10	86.67
*C. freundii*	90.95	100	94.29	93.81	93.81	93.81	93.81	93.81	93.81	91.43	92.38	92.86	91.90	90.95
*K. pneumoniae*	90.95	94.29	100	92.86	92.86	92.86	92.86	92.86	92.86	92.86	93.81	93.33	94.76	93.33
*S. typhimurium*	90.00	93.81	92.86	100	100	100	100	100	100	94.29	95.24	95.24	95.24	93.81
*S. enteritidis*	90.00	93.81	92.86	100	100	100	100	100	100	94.29	95.24	95.24	95.24	93.81
*S. typhi*	90.00	93.81	92.86	100	100	100	100	100	100	94.29	95.24	95.24	95.24	93.81
*S. paratyphi* A	90.00	93.81	92.86	100	100	100	100	100	100	94.29	95.24	95.24	95.24	93.81
*S. paratyphi* B	90.00	93.81	92.86	100	100	100	100	100	100	94.29	95.24	95.24	95.24	93.81
*S. paratyphi* C	90.00	93.81	92.86	100	100	100	100	100	100	94.29	95.24	95.24	95.24	93.81
*S. flexneri*	86.67	91.43	92.86	94.29	94.29	94.29	94.29	94.29	94.29	100	99.05	98.57	95.71	95.24
*S. boydii*	87.62	92.38	93.81	95.24	95.24	95.24	95.24	95.24	95.24	99.05	100	99.52	96.67	96.19
*S. sonnei*	88.10	92.86	93.33	95.24	95.24	95.24	95.24	95.24	95.24	98.57	99.52	100	96.19	95.71
*S. dysenteriae*	88.10	91.90	94.76	95.24	95.24	95.24	95.24	95.24	95.24	95.71	96.67	96.19	100	98.57
*E. coli* O157:H7	86.67	90.95	93.33	93.81	93.81	93.81	93.81	93.81	93.81	95.24	96.19	95.71	98.57	100

### Protein modeling

**Fig. 2:**
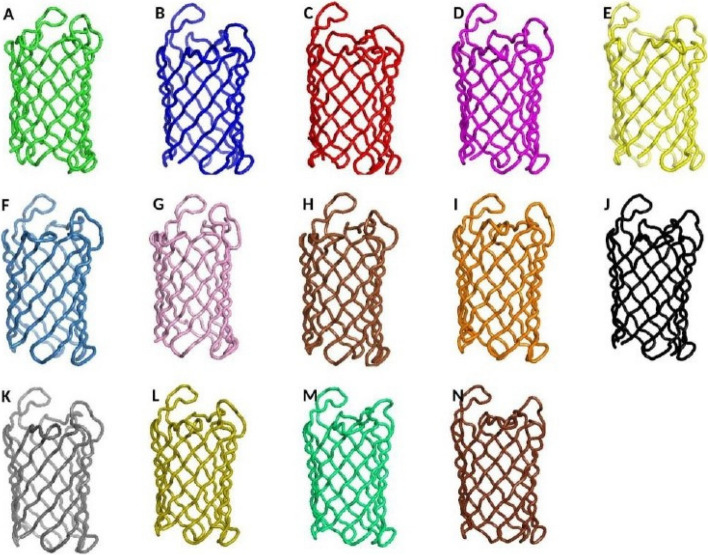
In silico modeled and refined three-dimensional structures of OmpL proteins of *Enterobacteriaceae* pathogens. **A** *Citrobacter freundii*. **B** *E coli* O 157:H7. **C** *Shigella boydii*. **D** *Shigella flexneri*. **E** *Shigella sonnei*. **F** *Shigella dysenteriae*. **G** *Salmonella enteritidis*. **H** *Salmonella typhimurium*. **I** *Salmonella typhi*. **J** *Salmonella paratyphi* A. **K** *Salmonella paratyphi* B. **L** *Salmonella paratyphi* C. **M** *Klebsiella pneumonia*. **N** *Klebsiella oxytoca*

Robetta server has generated the 3-D models of OmpL proteins for all the 14 *Enterobacteriaceae* pathogens. The Robetta server has generated five models of 3-D protein structure for each protein. Upon, the top protein structure model from each OmpL structure was further refined and then used for the prediction of discontinuous B-cell epitopes (Fig. 2). Protein structural refinement brings the structural quality of the protein proximate to the experimental preciseness. Therefore, the perfectly refined structure can be used for the prediction of discontinuous B-cell epitopes, which are formed as a result of protein folding and exposed on the structure of protein.

### Structural homology in the predicted structures of OmpL

While the sequence alignment is important to show the amino acid sequence homology in the protein for assessing the conservation of linear B-cell epitopes, structural alignment reveals the identity of confirmational B-cell epitopes that are formed as a result of protein folding. The percent identity matrix of OmpL protein structures is represented in Table 3. It is clear that the structural components of the OmpL are also highly conserved with > 90% which indicates the degree of conservation of discontinuous B-cell epitopes in the OmpL protein.

**Table 3:** Percent identity matrix representing the structural homology of the OmpL protein of Enterobacteriaceae pathogens

	*K. oxytoca*	*C. freundii*	*K. pneumoniae*	*S. typhimurium*	*S. enteritidis*	*S. typhi*	*S. paratyphi* A	*S. paratyphi* B	*S. paratyphi* C	*S. flexneri*	*S. boydii*	*S. sonnei*	*S. dysenteriae*	*E. coli* O157:H7
*K. oxytoca*	100	98	97	100	97	97	92	97	97	98	97	98	95	95
*C. freundii*	98	100	99	98	100	100	92	100	100	100	100	100	95	94
*K. pneumoniae*	97	99	100	97	99	99	92	100	99	100	100	99	96	94
*S. typhimurium*	100	98	97	100	97	97	92	97	97	97	97	97	98	94
*S. enteritidis*	97	100	99	97	100	99	92	99	100	100	100	100	96	94
*S. typhi*	97	100	99	97	99	100	92	99	100	100	100	100	96	94
*S. paratyphi* A	92	92	92	92	92	92	100	92	92	93	92	92	93	92
*S. paratyphi* B	97	100	100	97	99	99	92	100	100	100	100	99	95	94
*S. paratyphi* C	98	100	99	97	100	100	92	100	100	100	100	100	95	94
*S. flexneri*	98	100	100	97	100	100	93	100	100	100	100	100	97	94
*S. boydii*	97	100	100	97	100	100	92	100	100	100	100	100	96	94
*S. sonnei*	98	100	99	97	100	100	92	99	100	100	100	100	95	94
*S. dysenteriae*	95	95	96	98	96	96	93	95	95	97	96	95	100	97
*E. coli* O157:H7	95	94	94	94	94	94	92	94	94	94	94	94	97	100

### Linear B-cell epitopes in OmpL

**Fig. 3:**
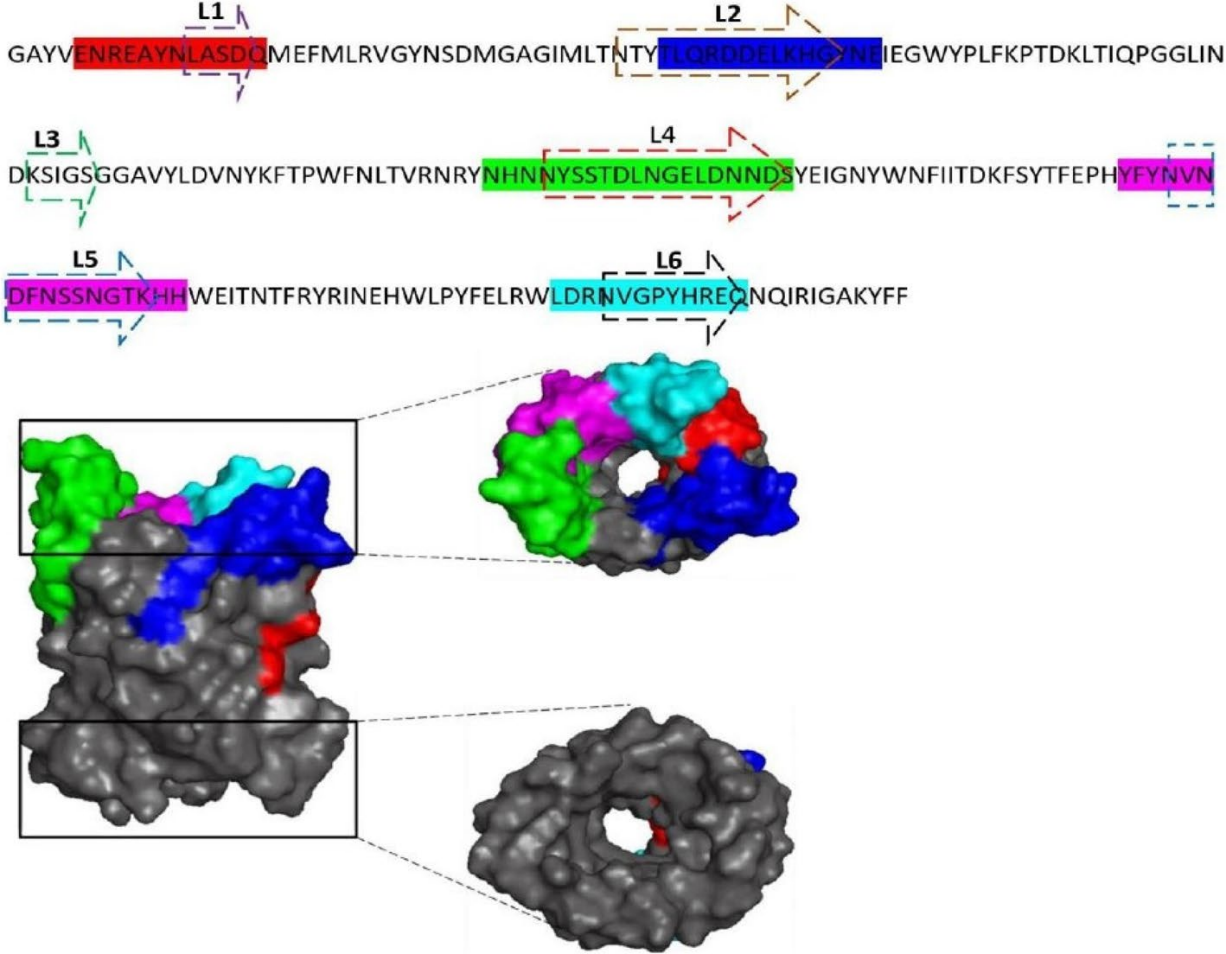
Linear B-cell epitopes predicted in the OmpL of *S. typhimurium*. The linear B-cell epitopes are located within the coordinates of the outer membrane loops of OmpL in the extracellular region

The OmpL protein contains predominant B-cell epitopes which are completely exposed out for the accessibility to antibodies. We predicted a total of five B-cell epitopes in the OmpL of *S. typhimurium* with the help of BepiPred tool (Table 4) (Fig. 3). Upon submission of amino acid sequence to BepiPred tool, the B-cell epitope peptide regions with a prediction score of greater than or equal to the default threshold value of 0.5 were chosen for further analysis. Previous study by Yang et al. has shown that the OmpL protein contains of six outer membrane loops [10]. In our observation, we clearly understood that the five linear B-cell epitopes ENREAYNLASDQ (25aa–36aa), TLQRDDELKHGYNE (60aa–73aa), NHNNYSSTDLNGELDNNDS (127aa–145aa), YFYNVNDFNSSNGTKHH (168aa–184aa), and LDRNVGPYHREQ (208aa–219aa) predicted in the OmpL of *S. typhimurium* are organized in these outer membrane loops which are clearly exposed out on the structure of protein (Fig. 3).

**Table 4:** Linear B-cell epitopes predicted in the OmpL protein of *S. typhimurium*

Epitope	Peptide region	Conservancy (%)
ENREAYNLASDQ	25–36	100
TLQRDDELKHGYNE	60–73	78.57
NHNNYSSTDLNGELDNNDS	127–145	78.94
YFYNVNDFNSSNGTKHH	168–184	64.70

### Confirmational/discontinuous B-cell epitopes in OmpL

**Fig. 4:**
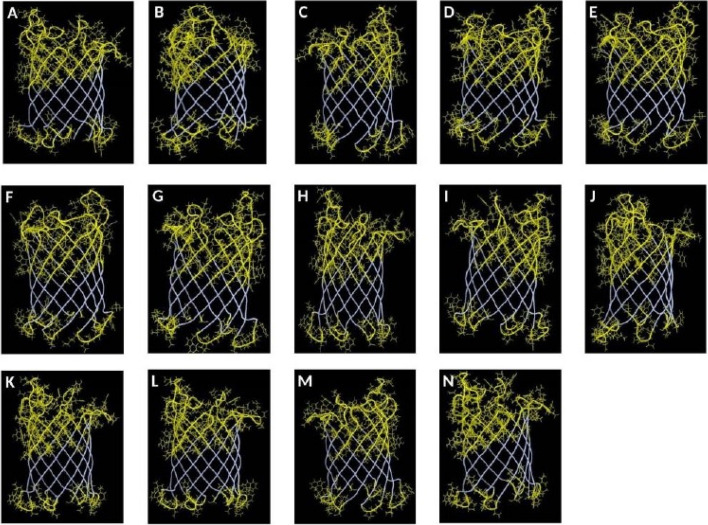
Discontinuous B-cell epitopes predicted in the OmpL protein of **A** *Citrobacter freundii*. **B** *E coli* O 157:H7. **C** *Shigella boydii*. **D** *Shigella flexneri*. **E** *Shigella sonnei*. **F** *Shigella dysenteriae*. **G** *Salmonella enteritidis*. **H** *Salmonella typhimurium*. **I** *Salmonella typhi*. **J** *Salmonella paratyphi* A. **K** *Salmonella paratyphi* B. **L** *Salmonella paratyphi* C. **M**. *Klebsiella pneumonia*. **N**. *Klebsiella oxytoca*. The discontinuous B-cell epitope regions are highlighted in the yellow regions of OmpL protein structures

Furthermore, discontinuous B-cell epitopes at various amino acid regions of OmpL structures from the 14 *Enterobacteriaceae *pathogens were also identified (Fig. 4). These discontinuous epitopes are peptide regions that are distributed along the protein sequence, which are recognized by antibodies when once they come close during protein folding. We identified several discontinuous B-cell epitopes in the exposed loops of OmpL (Fig. 4). In contrast, several amino acid regions in the periplasmic region were also identified as discontinuous B-cell epitopes.

### B-cell epitope conservation

**Fig. 5:**
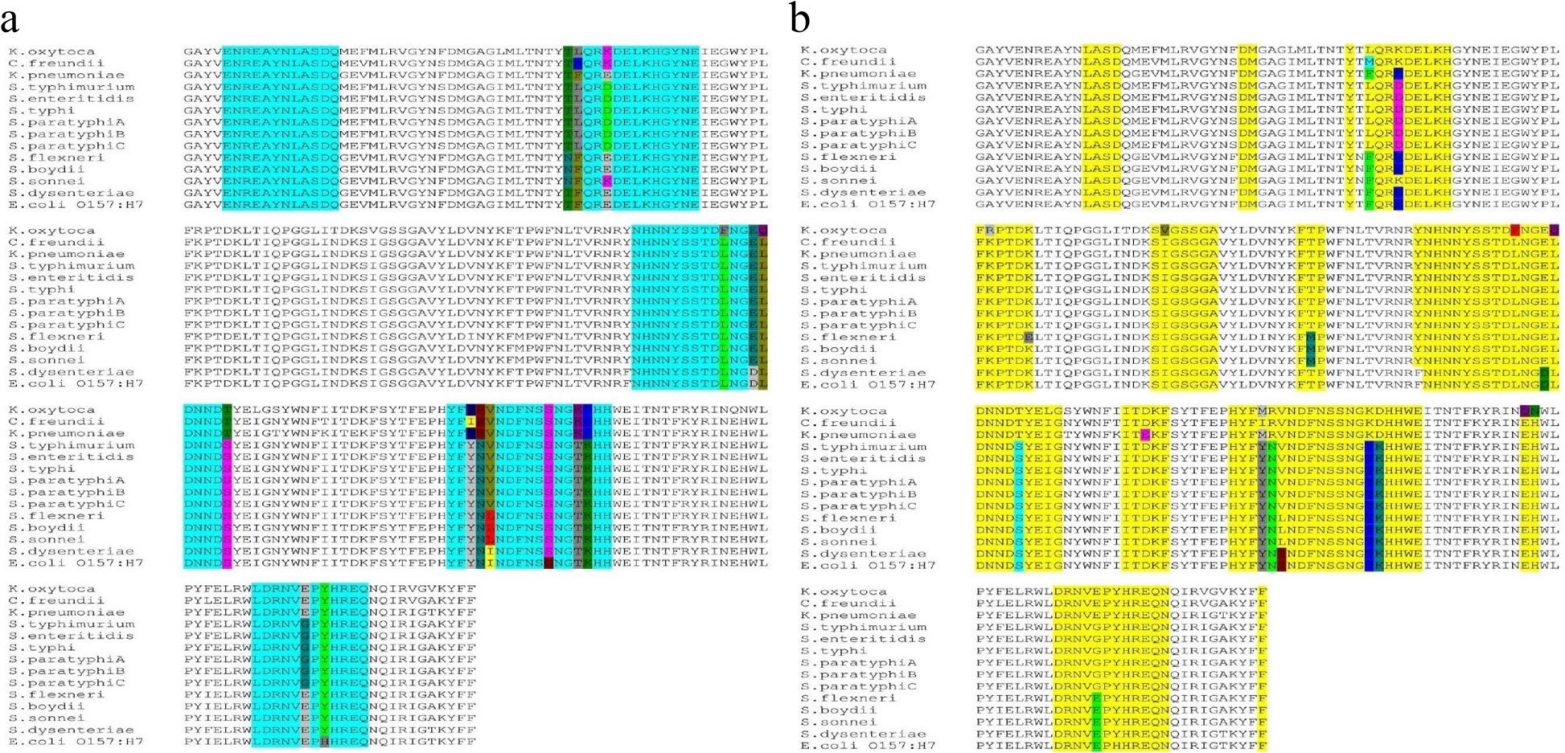
**a** Conservancy of the predicted linear B-cell epitopes in OmpL of *S. typhimurium* with the other *Enterobacteriaceae* members. The conservancy of linear B-cell epitopes ENREAYNLASDQ (25aa–36aa), TLQRDDELKHGYNE (60aa–73aa), HNNYSSTDLNGELDNNDS (127aa–145aa), YFYNVNDFNSSNGTKHH (168aa–184aa), and LDRNVGPYHREQ (208aa–219aa) is 100%, 78.57%, 78.94%, 64.70 and 83.33% respectively. **b** Conserved discontinuous B cell epitopes in the OmpL of *Enterobacteriaceae *pathogens. Conserved discontinuous B-cell epitopes are in the 32–35, 48–49, 59, 61–69, 81–86, 99–105, 114–116, 126–140, and 141

Both the linear and discontinuous B-cell epitopes were found to be highly conserved in the OmpL protein of *Enterobacteriaceae*. Among the five predicted B-cell epitopes in OmpL of *S. typhimurium*, four epitopes ENREAYNLASDQ (25aa–36aa), TLQRDDELKHGYNE (60aa–73aa), HNNYSSTDLNGELDNNDS (127aa–145aa), and LDRNVGPYHREQ (208aa–219aa) are highly conserved among the other *Enterobacteriaceae* pathogens with an amino acid conservancy of 100%, 78.57%, 78.94%, and 83.33%, respectively (Table 4) (Fig. 5a). Only one linear B-cell epitope YFYNVNDFNSSNGTKHH (168aa–184aa) is least conserved among the *Enterobacteriaceae* pathogens with 64.70%. In parallel, the conservancy was also seen in various discontinuous B-cell epitope residues at the amino acid level in alignment file of OmpL (Fig. 5b). The conservancy of discontinuous B-cell epitope regions was identified in the amino acid regions 32–35, 48–49, 59, 61–69, 81–86, 99–105, 114–116, 126–140, 141–149, 156–160, 167–186, 197–198, 209–220, and 230 of OmpL protein.

## Discussion

Outer membrane proteins (OMPs) of gram-negative bacteria are highly immunogenic in nature as they elicit strong immune response against the pathogens. In *Enterobacteriaceae*, the OMPs are highly conserved and share amino acid sequence homology to a greater extent [19]. Research findings from our previous studies [8, 9] have shown that the cross-reactivity of antibodies directed against the outer membrane proteins among the pathogens of *Enterobacteriaceae*. This indicates the conserved nature of antibody binding immunodominant epitopes in the OMPs. Immunogenicity of the crude OMPs was studied against the *Enterobacteriaceae* pathogens [8]. Understanding the nature of specific OMP antigens is more important for the development of recombinant sub-unit vaccines. Until date, only few outer membrane proteins have been explored for their vaccine potential against the *Enterobacteriaceae* pathogens [9–11]. The outer membrane porin (Omp) L is one such potential antigenic protein which was studied for its immunogenicity against salmonellosis in mice model [10]. The OmpL (also called as YshA) is a 230 amino acid long protein characterized by the presence of 12 β-trans membrane strands which expose 6 outer membrane loops into extracellular and 5 internal turns in periplasmic regions [10]. Furthermore, the OmpL protein is highly expressible in *E. coli* host expression system owing for its choice in the development of recombinant subunit vaccines. The recombinant version of OmpL protein (r-OmpL) was found to be highly immunogenic in eliciting immune response against the *S. typhimurium* and has shown 100% protection against the lethal challenge of 5 × 108 CFU/mL of *S. typhimurium*. Being highly conserved, the anti-OmpL sera have shown seroreactivity with the other serovars of *Salmonella* also. Additionally, in silico analysis has shown that all the outer membrane exposed loops in OmpL protein are extremely hydrophilic and completely exposed out. Importantly, phylogenetic analysis of OmpL protein has shown that the *Salmonella* serovars such as *typhimurium*, *enteritidis*, *typhi*, and *paratyphi* (A, B, and C) formed a monophyletic group and the other prominent *Enterobacteriaceae* pathogens *E. coli* O 157: H7, *Shigella* sp., *K. oxytoca*, and *C. koseri* as their close relatives with very less evolutionary divergence. These interesting findings have provoked us to analyze the OmpL protein for its amino acid and epitope conservancy among the *Enterobacteriaceae* pathogens. For the best of our knowledge, very few attempts were made to identify the conserved immunodominant epitopes in OMPs. In this study, using various bioinformatics resources, we determined the sequence homology in OmpL among a few pathogenic *Enterobacteriaceae* members with supreme focus on foodborne and extraintestinal infection outbreaks. The OmpL protein is highly conserved among the *Enterobacteriaceae* pathogens which signify the likelihood of the presence of conserved immunodominant B-cell epitopes (Table 2) (Fig. 1). The five linear B-cell epitopes which are predicted based on the amino acid sequence of OmpL of *S. typhimurium* are in the coordinates of outer membrane loops in extracellular region. Being hydrophilic, these loops are completely exposed out with immediate access of epitopes to the antibodies. The 4 linear B-cell epitopes ENREAYNLASDQ (25aa–36aa), TLQRDDELKHGYNE (60aa–373aa), NHNNYSSTDLNGELDNNDS (127aa–145aa), and LDRNVGPYHREQ (208aa–3219aa) are highly conserved among the other *Enterobacteriaceae* pathogens with high degree of sequence similarity in B-cell epitopes. Though there are few mutated positions in the amino acid residues of these B-cell epitopes, the higher sequence identity in the B-cell epitopes would vouch for the cross-reactivity as this case was observed in few studies [20, 21]. The discontinuous B-cell epitope regions predicted from the highly refined models of OmpL structures are also conserved among the *Enterobacteriaceae* pathogens. Unlike linear B-cell epitopes where the epitopes are in the extracellular loops, the discontinuous B-cell epitopes are also predicted in the intracellular regions of OmpL protein structures because the regions are open. But the antibodies will recognize only the surface-exposed epitopes since the periplasm regions are located within the cell wall of bacteria. Whatever the case may be, both linear and discontinuous B-cell epitopes are exposed out since they are in the extracellular loops and are highly conserved among the *Enterobacteriaceae* pathogens. Therefore, the anti-OmpL antibodies would likely prevent the adhesion of the *Enterobacteriaceae* pathogens *E. coli* O 157:H7, *Shigella* sp., *Salmonella* serovars, *Klebsiella* sp., and *C. freundii* to the epithelial cell wall and prevent their entry. Precisely, these immunodominant conserved epitopes can generate humoral immune response against the OmpL protein and activate B cells of different clones for conferring heterologous cross protection against the *Enterobacteriaceae *pathogens. From our analysis on the OmpL protein, we strongly suggest that the current research in recombinant vaccines against the *Enterobacteriaceae* pathogens can focus on targeting this potential immunodominant antigenic protein because it contains predominant conserved B-cell epitopes. Because the OmpL of *S. typhimurium* which we considered for analysis is highly conserved among other *Enterobacteriaceae* pathogens in terms of its sequence and structure, this protein can be used as a broad-spectrum recombinant subunit vaccine for the gastrointestinal infections caused by the *Enterobacteriaceae* pathogens. Similar to our previous reports on subunit vaccine development, the OmpL can be cloned and expressed and can be administered to mice along with the potential adjuvants like Freund’s adjuvants to induce strong immunogenicity [8, 9, 14].

Though this in silico study has proven the presence of highly conserved B-cell epitopes (linear and conformational), further in vitro validation is required to assess the antibody-mediated cross-reactivity towards the OmpL of different *Enterobacteriaceae* pathogens. Conceivably, the conserved B-cell epitopes will assure the antibody-mediated-cross-reactivity towards this potential antigenic protein which will provide clear insight into the development of a broad-spectrum subunit vaccine against enteric infections.

## Conclusion

The outer membrane proteins of *Enterobacteriaceae* are highly immunogenic and share sequence homology. This property resembles the presence of conserved immunodominant epitopes which are very crucial in generating heterologous immune response and confer protection against multiple pathogens simultaneously. We have identified such immunodominant conserved B-cell epitopes in the OmpL protein of major pathogenic representatives of *Enterobacteriaceae* such as *E. coli* O157:H7, *Shigella* sp., *Salmonella* sp., *Klebsiella* sp., and *C. freundii*. This computational evidence will be a promising call for current vaccine design and development which can generate heterologous immune response against these multiple *Enterobacteriaceae* pathogens and confer cross protection.

## Data Availability

Data sharing is not applicable to this article as no datasets were generated or analyzed during the current study.
